# Knowledge Gaps in Evidence-Based Extravasation Management: A Cross-Sectional Survey of Surgical Specialists in Türkiye

**DOI:** 10.3390/healthcare14213781

**Published:** 2026-11-05

**Authors:** Mert Gündoğdu, Nazım Canberk Kılıç, Samir Ilgaroğlu, Zeynep Akdeniz Doğan, Özgür Baysal

**Affiliations:** 1 Department of Orthopedics and Traumatology, Marmara University School of Medicine, Istanbul 34890, Türkiye; mertgntr@gmail.com (M.G.); opdrnckilic@gmail.com (N.C.K.); doktorsamir.zeynal@gmail.com (S.I.); 2 Department of Plastic, Reconstructive and Aesthetic Surgery, Marmara University School of Medicine, Istanbul 34854, Türkiye; zakdeniz@marmara.edu.tr

**Keywords:** extravasation injury, physician knowledge, patient safety, clinical decision-making, cross-sectional survey, vesicant agent

## Abstract

Background/Objectives: Severe extravasation injuries require prompt, agent-specific intervention to prevent avoidable tissue loss, yet they are often first assessed by surgeons with no structured training in their management. This study aimed to identify knowledge gaps in evidence-based extravasation management and to determine whether seniority, formal training or institutional factors are associated with a higher knowledge score. Methods: A cross-sectional web-based survey was conducted in June 2026 among orthopedic and plastic surgeons in Türkiye. In total, 359 eligible physicians responded to a 17-item knowledge instrument covering definitions, severity grading, treatment decisions and risk factors, alongside items on demographics, training and institutional infrastructure. Scores were dichotomized at 70% correct to define a high knowledge level, and logistic regression identified independent predictors. Results: The mean knowledge score was 10.0 ± 3.5 of 17 (KR-20 = 0.795), and 127 of 359 participants (35.4%) reached the high-knowledge threshold. Deficiencies were not uniformly distributed: accuracy was high for basic definitional items (76–90%) but markedly lower for time-sensitive therapeutic decisions, including antidote selection and flush-technique indications (51–59%), and for recognition of high-risk agents (24.0%). No independent association was detected between knowledge level and professional seniority, formal training or institution type. More frequent case exposure was associated with a higher mean score in unadjusted analysis (*p* = 0.017) but was not retained after adjustment. Conclusions: Substantial knowledge gaps were identified across surgical specialties, concentrated in the time-sensitive decisions in which errors may contribute to preventable tissue loss. No independent association was detected between knowledge level and either seniority or formal training; experiential learning alone may therefore not ensure familiarity with current evidence-based management. Standardized cross-specialty education and institutional protocols are needed.

## 1. Introduction

Extravasation is the inadvertent leakage of intravenously administered fluids or medications into the surrounding soft tissues following failure of a vascular access device. Although many cases result in only mild local reactions, severe extravasation may lead to progressive soft-tissue necrosis, compartment syndrome, tendon or nerve injury, permanent functional impairment, and occasionally limb-threatening complications. The extent of tissue damage is influenced by the physicochemical properties of the extravasated agent, the volume infiltrated, the anatomical site involved, and patient-related factors [1,2,3,4].

As intravenous therapy is routinely used across virtually all medical specialties, extravasation remains an important iatrogenic complication encountered in emergency departments, intensive care units, operating rooms, oncology services, radiology departments, and general hospital wards. While prevention remains the most effective strategy, clinical outcomes largely depend on the appropriateness of the initial therapeutic decisions made immediately after the injury occurs. Early discontinuation of the infusion, appropriate limb positioning, correct selection of warm or cold application according to the causative agent, timely administration of specific antidotes, consideration of saline flush-out techniques when indicated, and prompt surgical consultation for severe injuries are all essential components of evidence-based management. Delayed or inappropriate interventions during the first hours after extravasation may result in avoidable tissue loss, complex reconstructive procedures, prolonged hospitalization, and increased healthcare costs [5,6,7,8].

Unlike many surgical conditions in which definitive treatment can be planned after specialist consultation, successful management of extravasation often depends on decisions made by the first physician evaluating the patient. These decisions are highly time-sensitive and require familiarity with specific management algorithms rather than general surgical knowledge alone. Errors in antidote selection, inappropriate application of warm or cold therapy, failure to recognize candidates for saline flush-out, or delayed referral for surgical evaluation may substantially worsen patient outcomes.

Despite the publication of international guidelines, little is known about surgeons’ familiarity with current evidence-based recommendations for extravasation management. Existing studies have primarily focused on epidemiology, treatment techniques, or institutional practices, whereas clinicians’ knowledge of time-critical therapeutic decisions has received comparatively little attention. Because successful management relies on a sequence of evidence-based interventions rather than a single therapeutic measure, deficiencies in clinicians’ knowledge may directly influence patient outcomes. Identifying these deficiencies is therefore an important step toward improving patient safety, guiding targeted educational initiatives, and promoting standardized institutional management pathways.

Accordingly, the primary aim of this study was to identify specific knowledge gaps in evidence-based extravasation recognition and management among surgical specialists who may be consulted for severe tissue injury in clinical practice. A secondary objective was to compare knowledge patterns between orthopedic and traumatology surgeons and plastic, reconstructive, and aesthetic surgeons, given that both specialties may be involved in the management of established extravasation injuries—particularly in settings where dedicated wound-care teams are unavailable—and to examine whether professional seniority or prior structured training was associated with better performance. We hypothesized that important deficiencies would exist in time-critical aspects of extravasation management, particularly regarding antidote selection, local treatment strategies, saline flush-out indications, and indications for urgent surgical consultation.

## 2. Materials and Methods

This study was designed as a cross-sectional, descriptive, web-based survey aimed at evaluating the clinical experience, institutional infrastructure, and knowledge of evidence-based extravasation management among orthopedic and traumatology surgeons (including hand surgeons) and plastic, reconstructive, and aesthetic surgeons practicing in Türkiye. Physicians and resident physicians working in university hospitals, training and research hospitals, state hospitals, private hospitals, and private practices across the country were invited to participate on a voluntary basis. No personal identifying information was collected, and anonymity was strictly maintained.

### 2.1. Questionnaire Design

The questionnaire was developed following an extensive literature review and current international guidelines [8,9,10,11]. Each keyed correct answer was adjudicated against these sources; an item-by-item mapping of every knowledge item to its source, with the verbatim supporting text, is provided in Table S1c [3,5,12,13,14,15,16]. Draft items were then reviewed by surgeons experienced in extravasation management to ensure clinical relevance and clarity before online distribution. The survey consisted of 17 knowledge items addressing definitions and recognition, severity grading according to the Infusion Nurses Society (INS) classification, treatment decisions, indications for urgent consultation, and risk factors for extravasation injury, of which 13 were single-best-answer items and 4 were multiple-answer items. Each item contributed a maximum of one point, giving a total score range of 0–17. Multiple-answer items were scored on an all-or-nothing basis: an item was counted as correct only when every correct option was selected and no distractor option was marked, and no partial credit was awarded. One multiple-answer item (clinical features of INS Grade 4) contained four correct options and no distractor, so that a correct response required selection of all four. Unanswered items were scored as incorrect. Additional items evaluated participant demographics, clinical experience, prior training exposure, sources of knowledge acquisition, institutional infrastructure, and management difficulties (Tables S1, S1b and S1c).

Although formal psychometric validation (including construct validity and test–retest reliability) was not performed, the questionnaire underwent expert review for face validity before dissemination. The draft items and the answer key were reviewed independently by two orthopaedic surgeons experienced in the management of extravasation injury, who assessed each item for clinical relevance, clarity and consistency with the source guidelines; disagreements were resolved by discussion until consensus was reached, and no formal measure of inter-reviewer agreement was calculated. The instrument was not pilot-tested or cognitively interviewed before dissemination. It should be noted that the expert review panel consisted of surgeons from one of the two specialties studied; independent multidisciplinary validation was not performed, which may have introduced specialty-specific perspective in item formulation. Internal consistency was subsequently assessed using the Kuder–Richardson Formula 20 (KR-20), which yielded an acceptable coefficient of 0.795.

### 2.2. Data Collection

**Figure 1:**
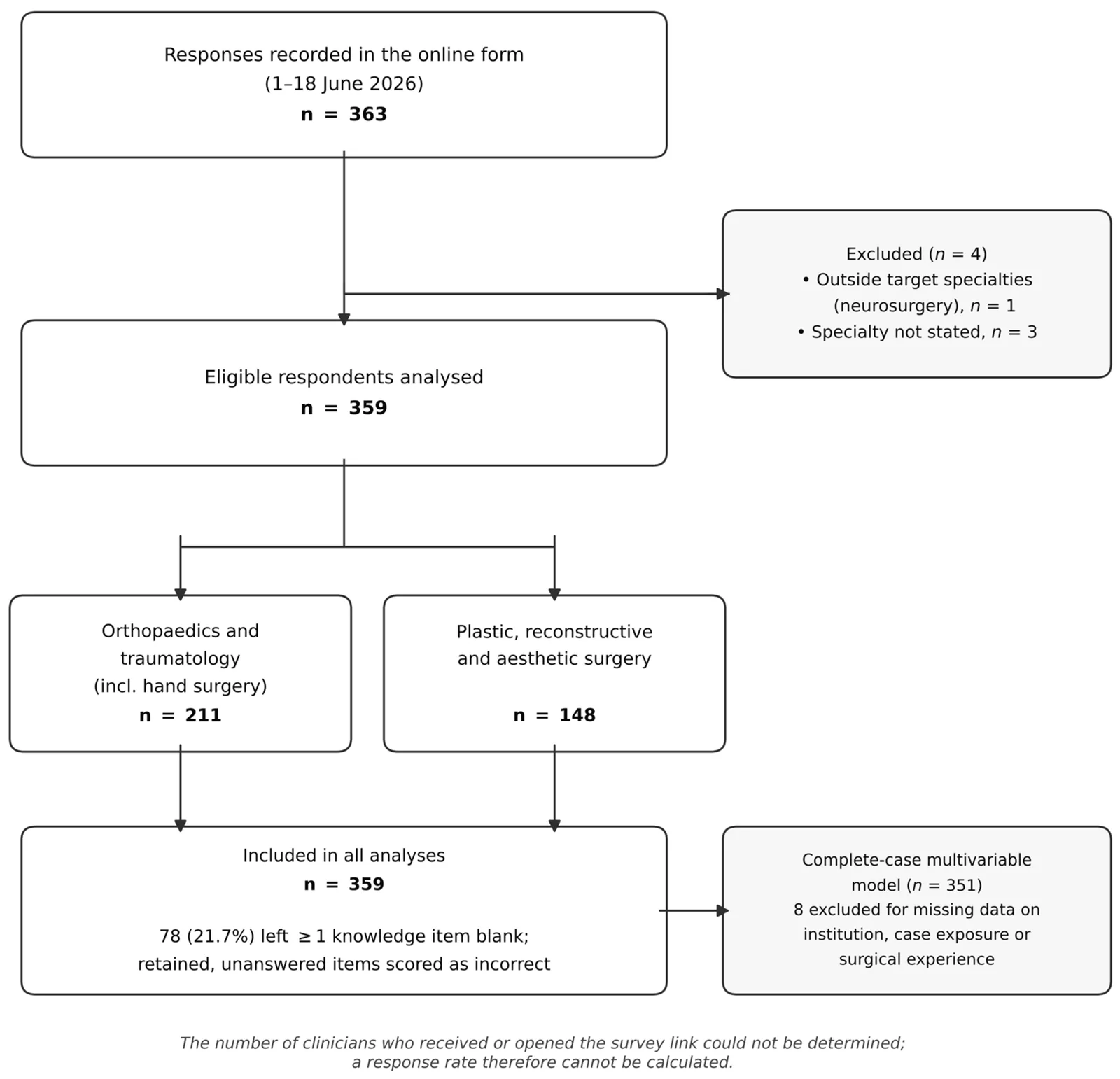
Participant flow. Of 363 responses recorded between 1 and 18 June 2026, four were excluded (one respondent from outside the target specialties and three who did not state a specialty), leaving 359 for analysis. The multivariable model was fitted on 351 complete cases.

The survey was distributed through the e-mail lists and instant messaging groups of the two relevant specialty associations in Türkiye, which are used by orthopaedic and traumatology surgeons and by plastic, reconstructive and aesthetic surgeons, between 1 and 18 June 2026. Recruitment relied on convenience and snowball sampling: members were free to forward the link to colleagues, so the number of clinicians who received or opened the invitation could not be determined and a response rate cannot be calculated. Eligibility was restricted to physicians practising in Türkiye in orthopaedics and traumatology or in plastic, reconstructive and aesthetic surgery, at any stage of training. Of the 363 responses recorded, 4 were excluded (1 respondent from outside the target specialties and 3 who did not state a specialty), leaving 359 for analysis (Figure 1). Recruitment was open to eligible clinicians practising anywhere in Türkiye, but the sample was not drawn by probability methods and is not a nationally representative sample of either specialty. Partially completed questionnaires were retained; unanswered knowledge items were scored as incorrect. The questionnaire was administered via Google Forms, with the single-response restriction enabled to prevent duplicate submissions from the same device. Participation was voluntary, anonymous, and without financial incentive. Participants were encouraged to share the survey with eligible colleagues, resulting in a non-probability convenience sample obtained through an online snowball recruitment strategy. Because recruitment relied on self-referral through online platforms, several methodological limitations should be acknowledged: verification of participants’ professional credentials was not performed beyond self-reported specialty and institutional affiliation; completion time was not recorded, and the use of external resources—including internet searches or artificial intelligence tools—during questionnaire completion cannot be excluded; and although the single-response restriction was enabled at the device level, forwarding of the survey link beyond the intended target population cannot be fully ruled out. These factors introduce the possibility of selection bias and response validity concerns and are acknowledged as limitations of the study.

### 2.3. Sample Size

No a priori sample-size calculation was performed. Recruitment was open to all eligible clinicians during the study period, and the achieved sample of 359 participants was determined by the number of voluntary responses received. To indicate the precision of the achieved sample, an approximate detectable-effect calculation was carried out after data collection using the standard normal approximation for the comparison of two independent proportions. With 211 orthopaedic and 148 plastic surgeons, a two-sided alpha of 0.05 and 80% power, the study was able to detect an absolute difference of approximately 15 percentage points (14.8) in the correct-response rate for a single dichotomous knowledge item between the two specialty groups, assuming a baseline correct-response rate of 50% in the orthopaedic group. This figure refers to a single item and is reported as a descriptive indication of precision rather than as a sample-size justification; the study was not designed around a single primary outcome.

### 2.4. Statistical Analysis

Statistical analysis was performed using IBM SPSS Statistics version 26.0 (IBM Corp., Armonk, NY, USA). As all study variables were categorical, descriptive statistics were reported as frequencies (n) and percentages (%). Responses to knowledge items were coded as “correct” or “incorrect”; for multiple-answer items, a response was accepted as correct only when all correct options were selected and no distractor option was marked.

Item-specific response patterns were compared across groups defined by specialty, professional seniority, and training status using the Pearson chi-square test. Where the expected cell frequency was below 5 in any cell of a 2 × 2 table, or in more than 20% of cells in larger contingency tables, Fisher’s exact test or the Fisher–Freeman–Halton extension was used, as appropriate. All tests were two-sided, and a *p* value < 0.05 was considered statistically significant. Variables with *p* < 0.20 in univariable analyses together with clinically relevant variables (specialty, professional seniority, formal training, institution type, frequency of encountering extravasation cases, and previous experience with surgically managed extravasation injuries) were entered into the multivariable logistic regression model.

Item-level comparisons of the 17 knowledge items across specialty, professional seniority and training status were pre-specified as exploratory. Because 17 comparisons were performed within each stratifying variable, *p* values were adjusted using the Holm procedure, and both unadjusted and adjusted values are reported in the Supplementary Tables.

Formal training was defined as any prior theoretical and/or practical training in extravasation management (n = 107) versus no formal training (n = 252); the three-category breakdown used in the item-level analysis is nested within this binary variable. Frequency of case exposure was dichotomised as frequent (at least a few times per month, n = 172) versus infrequent (less often, n = 187), and experience of surgical intervention as any versus none. The original response categories are reported in the Results, and the dichotomised variables were used in the subgroup comparisons and in the regression model.

### 2.5. Questionnaire Scoring

In addition to item-level analyses, an overall knowledge score (0–17) was calculated for each participant. Normality of score distribution was assessed using the Kolmogorov–Smirnov test together with histogram inspection. Continuous knowledge scores were summarized as mean ± standard deviation (SD) and median (interquartile range [IQR]) and compared using the Mann–Whitney U test or Kruskal–Wallis test, as appropriate.

For multivariable analysis, the overall knowledge score was dichotomized using a prespecified threshold of ≥70% correct answers (≥12/17), defining participants with a “high knowledge level”. This threshold was specified before the analysis and reflects the conventional pass mark used in postgraduate medical knowledge assessment; it does not represent a validated standard of clinical competence. Binary logistic regression (Enter method) was performed to identify independent predictors of high knowledge. Variables entered the model included specialty, professional seniority, formal training, institution type, frequency of encountering extravasation cases, and previous experience with surgically managed extravasation injuries. Results were reported as odds ratios (ORs) with 95% confidence intervals (CIs).

Unanswered knowledge items were scored as incorrect so that all item-level and total-score percentages are calculated from the full analytic sample (n = 359). A total of 428 knowledge responses were left blank, and 78 of the 359 participants (21.7%) did not answer at least one knowledge item. For the multivariable model, complete-case analysis was used: eight participants were excluded because of missing data on type of institution (n = 4), frequency of case exposure (n = 1) or experience of surgical intervention (n = 4), with one participant missing two of these variables, leaving 351 participants and 124 events.

Model performance was evaluated using the Hosmer–Lemeshow goodness-of-fit test, Nagelkerke’s R^2^, and the area under the receiver operating characteristic curve (AUC). Collinearity was assessed using variance inflation factors, and influential observations were examined using leverage values, standardised Pearson residuals and Cook’s distance. To examine the robustness of the findings, the multivariable model was repeated at four alternative thresholds for defining a high knowledge level—≥10/17 (the median), ≥11/17, ≥12/17 and ≥13/17—on the same complete-case set and with identical covariates (Table S7).

### 2.6. Self-Reported Data and Bias Considerations

All data on clinical experience, institutional infrastructure, and case encounters were based solely on participants’ self-reports and were therefore subject to recall bias and social desirability bias. No independent verification against institutional records was performed.

## 3. Results

### 3.1. Participant Characteristics

A total of 359 physicians participated, most of whom were orthopedic surgeons and resident physicians working in university or training-research hospitals (Table 1).

### 3.2. Training Status and Institutional Infrastructure

Fewer than one in five participants (19.5%) had received both theoretical and practical training in extravasation management, and nearly half had learned solely through clinical experience. Most encountered extravasation cases only occasionally, and surgical intervention was rarely required (Table 1).

### 3.3. Sources of Knowledge, Consultation Patterns, and Institutional Infrastructure

Knowledge was acquired predominantly through clinical experience and residency training rather than guidelines or institutional protocols. Non-cytotoxic agent extravasation and suspected compartment syndrome were the most common consultation scenarios, and delayed recognition of the injury was the most frequently cited management difficulty (Table 2).

Half of participants reported no written institutional protocol for extravasation management, and most institutions lacked a functioning incident-reporting system. The large majority of participants expressed a desire for further training (Table 2).

### 3.4. Responses to Knowledge Items

Participants performed well on items assessing basic definitions and recognition (correct response rates of 76–90%) but considerably worse on items requiring selection of all correct options among multiple answers (14–27%) and on items assessing pharmacologic and treatment decisions (51–59%) (Table 3).

Option-level analysis clarified this pattern: individual correct components of the multiple-answer items were often recognized at moderate-to-high rates, but requiring all components to be selected simultaneously—with no distractor marked—sharply reduced the overall correct rate. This effect was most pronounced for the four features of INS Grade 4, none of which was recognized by more than 59% of participants individually, yielding an all-correct rate of only 13.9%. For peripheral IV catheterization risk factors, the incorrect option was also selected by over one-fifth of participants, further lowering the all-correct rate (Table S2).

### 3.5. Overall Knowledge Score

The mean overall knowledge score was 10.0 ± 3.5 (median, 10; IQR, 8–13; range, 0–17), corresponding to an overall correct response rate of 58.8%. The questionnaire demonstrated acceptable internal consistency (KR-20 = 0.795). Using the predefined threshold of ≥70% correct answers (≥12/17), 127 participants (35.4%) were classified as having a high level of knowledge.

Plastic surgeons achieved significantly higher overall knowledge scores than orthopedic surgeons (11.1 ± 3.3 vs. 9.2 ± 3.5, respectively; Mann–Whitney U test, *p* < 0.001). Likewise, the proportion of participants classified as having a high level of knowledge was significantly greater among plastic surgeons than orthopedic surgeons (49.3% vs. 25.6%, *p* < 0.001) (Table 4).

Participants who encountered extravasation cases more frequently demonstrated significantly higher overall knowledge scores than those with less frequent exposure (*p* = 0.017), although the proportion with a high knowledge level did not differ significantly (*p* = 0.072). No significant associations were observed between overall knowledge score and professional seniority, formal training, institution type, or previous experience with surgically managed extravasation injuries (all *p* > 0.05) (Table 4).

### 3.6. Predictors of High Knowledge Level

In univariable logistic regression analysis, plastic surgery specialty (OR 2.83, 95% CI 1.81–4.42; *p* < 0.001), employment at a university or training and research hospital (OR 2.01, 95% CI 1.08–3.75; *p* = 0.029), and ≥10 years of professional seniority (OR 0.56, 95% CI 0.33–0.95; *p* = 0.031) were associated with high knowledge level. After adjustment for specialty, seniority, formal training, institution type, frequency of encountering extravasation cases, and previous surgical experience, plastic surgery remained the only independent predictor of high knowledge level (adjusted OR 3.00, 95% CI 1.74–5.18; *p* < 0.001). Neither formal training, professional seniority, institution type, frequency of case exposure, nor previous surgical experience remained independently associated with knowledge level (Table 5). The multivariable model demonstrated acceptable calibration (Hosmer–Lemeshow *p* = 0.616) but limited discriminative ability (Nagelkerke R^2^ = 0.100; AUC = 0.662) indicating that additional unmeasured factors probably contribute to physicians’ knowledge levels.

### 3.7. Sensitivity Analysis

Sensitivity analysis using the median knowledge score (≥10/17) as an alternative threshold yielded similar results. Plastic surgery specialty remained the only independent predictor of higher knowledge (adjusted OR, 2.65; 95% CI, 1.55–4.55; *p* < 0.001), whereas none of the remaining variables reached statistical significance (Table S3). These findings indicate that the association between specialty and knowledge level was robust and independent of the selected cut-off value. Repeating the model across all four thresholds (≥10/17, ≥11/17, ≥12/17 and ≥13/17) gave the same picture: specialty was the only covariate significantly associated with a high knowledge level at every threshold (adjusted OR 2.35–3.33; *p* ≤ 0.001 throughout), no other covariate reached significance at any threshold, and model performance was essentially unchanged (Nagelkerke R^2^, 0.089–0.102; AUC, 0.651–0.669) (Table S7).

### 3.8. Comparison by Specialty

Plastic surgeons achieved higher correct-response rates than orthopedic surgeons on several knowledge items, but after Holm adjustment for the 17 item-level comparisons only three differences remained significant, all of them pharmacologic treatment decisions: the antidote for anthracycline extravasation (72.3% versus 36.5%; adjusted *p* < 0.001), the antidote for vasopressor extravasation (68.2% versus 48.3%; adjusted *p* = 0.003), and the decision between warm and cold application (70.3% versus 50.7%; adjusted *p* = 0.003). No other item differed significantly after adjustment (Table S4).

### 3.9. Comparison by Professional Seniority

When participants were stratified into four groups by professional seniority (0–2 years, 3–5 years, 6–10 years, and ≥10 years), one item differed significantly after Holm adjustment: knowledge of the antidote for anthracycline extravasation declined with increasing seniority (57.3%, 59.1%, 51.9%, and 33.7% for 0–2, 3–5, 6–10, and ≥10 years, respectively; adjusted *p* = 0.026). No other item differed significantly between seniority groups (Table S5).

### 3.10. Comparison by Training Status

No knowledge item differed significantly between training-status categories after Holm adjustment. The item on information that should not be included in documentation showed a nominally significant difference (77.1%, 54.1%, and 72.6% for theoretical and practical training, theoretical training only, and no formal training, respectively; unadjusted *p* = 0.034), but this did not survive adjustment for multiple comparisons and should be regarded as exploratory (Table S6).

## 4. Discussion

Extravasation is an uncommon but potentially devastating complication in which patient outcomes largely depend on the appropriateness of early management. The present survey demonstrated substantial knowledge gaps among surgical specialists regarding evidence-based extravasation management, despite their potential role in managing severe extravasation injuries requiring consultation. Critically, these deficiencies were not uniformly distributed across knowledge domains but were concentrated in time-sensitive clinical decision-making—precisely the areas in which errors are most likely to result in avoidable tissue loss. Although participants demonstrated satisfactory familiarity with basic concepts such as the definition of extravasation and the general role of surgical consultation in patient management, considerably lower performance was observed for questions involving therapeutic decision-making and recognition of high-risk clinical situations. These findings are clinically important because successful extravasation management depends on rapid recognition and timely implementation of appropriate interventions during the early stages of injury.

Particularly concerning was the limited knowledge regarding high-risk extravasation injuries. Only one-quarter of participants correctly identified all characteristics of high-risk extravasation agents [16], while fewer than 15% correctly recognized all clinical features of INS Grade 4 extravasation. Likewise, knowledge of peripheral intravenous catheter-related risk factors was poor. Failure to recognize these high-risk situations could delay diagnosis and appropriate treatment and may therefore represent a potential patient-safety vulnerability, although this survey measured questionnaire responses rather than clinical decisions or patient outcomes [11,17,18,19].

The most clinically relevant finding of our study was the limited knowledge regarding treatment algorithms. Approximately half of participants correctly identified the recommended antidotes for vasopressor and anthracycline extravasation, the appropriate use of warm versus cold application, and the indications and timing of the Gault saline flush-out technique [13,14]. Furthermore, fewer than one-third correctly recognized situations requiring urgent surgical consultation. These findings are particularly important because many therapeutic interventions in extravasation management are highly time dependent. Delayed administration of antidotes, inappropriate local treatment, or failure to recognize patients requiring early surgical evaluation could contribute to greater tissue damage and increased reconstructive requirements, although the present design cannot demonstrate that the observed knowledge scores caused such outcomes. Notably, the greatest deficiencies were observed in time-critical therapeutic decisions rather than in basic theoretical concepts. This finding suggests that future educational initiatives should emphasize practical, case-based training focused on evidence-based management algorithms rather than relying solely on conventional didactic teaching. Rather than reflecting isolated deficiencies, these results suggest that many surgeons are unfamiliar with key components of contemporary evidence-based extravasation management [12,20,21,22,23].

Another important observation was that no independent association was detected between professional seniority and knowledge level. More frequent case exposure was associated with a higher mean knowledge score in unadjusted analysis (*p* = 0.017), but this association was not retained in the adjusted model and the proportion reaching a high knowledge level did not differ significantly; experience therefore cannot be said to be unrelated to knowledge in every analysis, only that it was not an independent predictor here. Although many participants had encountered extravasation cases during clinical practice, structured education remained uncommon, and most respondents reported acquiring their knowledge primarily through clinical experience rather than formal teaching or guideline-based education. Nearly 90% also expressed a desire for additional training. Similar findings were observed in our previous survey evaluating knowledge of pneumatic tourniquet use among orthopedic surgeons, suggesting that clinical exposure alone may be insufficient to maintain up-to-date knowledge in patient safety-related topics. These findings support the need for structured educational programs incorporating current evidence and practical simulation-based training [24].

Although specialty comparison was a secondary objective of this study, plastic surgeons achieved higher correct-response rates on three pharmacological treatment-decision items that remained significant after adjustment for multiple comparisons. We did not measure case mix, curriculum content, referral volume or professional role in sufficient detail to test why this difference arose, and the following explanations are therefore offered as hypotheses rather than established mechanisms. Greater clinical exposure to complex soft-tissue injury and reconstructive procedures may contribute, as may differences in residency curricula and in the role each specialty plays in the referral pathway. Residual confounding cannot be excluded, and some items may align more closely with the routine practice of one specialty than the other; the specialty itself should not be interpreted as a cause of greater knowledge. Nevertheless, the overall pattern of results indicates that clinically important knowledge deficiencies were present in both specialties—most notably in antidote selection, recognition of INS Grade 4 features, and indications for urgent surgical consultation. These findings suggest that the knowledge gaps identified in this study are not primarily a function of specialty training alone but reflect the limited emphasis placed on extravasation management in surgical education more broadly. Since early recognition and initial management often occur before reconstructive surgeons become involved, familiarity with evidence-based extravasation management should be considered an essential competency for all physicians who may encounter these injuries [25,26].

Beyond individual knowledge deficits, our study also identified important institutional shortcomings. Nearly half of participants reported the absence of written institutional protocols, and incident-reporting systems were unavailable or rarely used in most centers. Similar inconsistencies in institutional practice have been reported previously despite the existence of published recommendations [27]. These findings suggest that improving extravasation management requires not only physician education but also wider implementation of standardized institutional protocols and multidisciplinary management pathways.

Interestingly, previous structured training was not independently associated with higher knowledge after adjustment for specialty and other covariates. This finding suggests that the mere presence of previous training may not be sufficient; rather, the quality, standardization, and recency of educational activities may be more important determinants of knowledge. It is also possible that existing educational programs emphasize theoretical concepts rather than practical, guideline-based decision making. Future educational interventions should therefore focus on standardized, competency-based training accompanied by periodic updates.

Several limitations should be acknowledged. Participation was voluntary and internet-based, introducing the possibility of selection bias. Because recruitment relied on convenience and snowball sampling through association mailing and messaging groups, clinicians who are more engaged with professional networks or more interested in this topic, may be over-represented; the findings therefore describe the respondents rather than the national population of orthopaedic and plastic surgeons and should be generalised with caution. Clinical experience and institutional practices were self-reported and therefore subject to recall and social desirability bias. The cross-sectional design precludes causal inference, and the questionnaire assessed theoretical knowledge using hypothetical scenarios and therefore may not reflect actual physician behavior or clinical performance in real-world practice. The instrument underwent expert review and showed acceptable internal consistency (KR-20 = 0.795), but internal consistency is not evidence of content, construct or criterion validity, and no formal psychometric validation, pilot testing or test–retest assessment was undertaken. The answer key was adjudicated against current guidelines by two orthopaedic surgeons; no plastic surgeon or non-surgical specialist took part in the review, and independent review by a wider multidisciplinary panel—for example, infusion therapy, oncology or pharmacy, and emergency or critical care—would have strengthened content validity, and the total score should therefore not be regarded as a validated measure of clinical competence. The threshold used to define a high knowledge level was investigator-defined, and dichotomising a 0–17 score discards information; the continuous score was therefore analysed alongside the binary outcome, and the multivariable model was repeated across four thresholds spanning ≥10/17 to ≥13/17, with consistent findings throughout (Table S7). Item-level subgroup comparisons were exploratory and are reported with Holm-adjusted *p* values. Finally, although the survey platform restricted multiple submissions from the same device, this does not guarantee a single response per respondent, consultation of external resources during completion could not be prevented, and professional credentials could not be independently verified. Unanswered knowledge items were scored as incorrect, which may underestimate true knowledge in the 78 participants (21.7%) who left at least one item blank.

## 5. Conclusions

This survey demonstrates that although surgeons are familiar with the fundamental concepts of extravasation, important deficiencies remain in several time-critical aspects of evidence-based management, including recognition of high-risk injuries, antidote selection, warm versus cold application, flush-out techniques, and indications for urgent surgical consultation. Because these decisions are time-critical, improving familiarity with current management algorithms warrants priority during residency training and continuing medical education. Standardized institutional protocols, multidisciplinary educational initiatives, and regular guideline updates are proposed as implications for evaluation rather than as interventions whose effectiveness is established by this study; whether they improve recognition, management or patient outcomes remains to be tested. Future multicenter studies evaluating whether structured educational interventions improve physician performance and patient outcomes are warranted.

## Figures and Tables

**Table 1:** Specialty, professional seniority and type of institution of the participants (n = 359).

Characteristic	n	%
Specialty		
Orthopaedics and Traumatology (incl. hand surgery)	211	(58.8)
Plastic, Reconstructive and Aesthetic Surgery (incl. other)	148	(41.2)
Professional seniority		
0–2 years (resident)	103	(28.7)
3–5 years (resident)	115	(32.0)
6–10 years (specialist)	52	(14.5)
≥10 years (senior specialist)	89	(24.8)
Type of institution		
University hospital	165	(46.0)
Training and research hospital	123	(34.3)
Private hospital	40	(11.1)
State hospital	17	(4.7)
Other (private office, city hospital, medical centre, retired)	10	(2.8)
Not specified	4	(1.1)

Percentages are calculated from the total analytic sample (n = 359) and rounded to one decimal place; columns may not sum to exactly 100.0 because of rounding. Four participants did not state their institution.

**Table 2:** Prior training, sources of knowledge, clinical exposure, institutional infrastructure and attitudes (n = 359).

Characteristic	n	%
Prior training in extravasation management		
Yes—both theoretical and practical	70	19.5
Yes—theoretical only	37	10.3
No structured training; learned through experience	175	48.7
No training at all	77	21.4
Sources of knowledge (multiple responses permitted)		
Medical school/residency training	219	61.0
Clinical experience	267	74.4
Academic articles and guidelines	93	25.9
Congress/symposium	39	10.9
Learning from colleagues	183	51.0
Institutional protocols	29	8.1
Frequency of encountering extravasation cases		
More than once per week	42	11.7
Once per week	31	8.6
A few times per month	99	27.6
A few times per year	169	47.1
Never encountered	17	4.7
Not specified	1	0.3
Proportion of cases requiring surgical intervention		
Majority of cases (>50%)	20	5.6
About half of cases (25–50%)	33	9.2
A minority of cases (<25%)	216	60.2
Never required	86	24.0
Not specified	4	1.1
Written institutional protocol for extravasation management		
Yes—exists and actively used	20	5.6
Yes—exists but underused	14	3.9
No institutional protocol	177	49.3
Do not know	147	40.9
Not specified	1	0.3
Incident reporting after an extravasation event		
Completed for every case	33	9.2
Completed for severe/surgical cases	47	13.1
Rarely completed	65	18.1
No reporting system, or not used	207	57.7
Not specified	7	1.9

Percentages are calculated from the total analytic sample (n = 359). For the multiple-response item (sources of knowledge) percentages sum to more than 100 because participants could select several options.

**Table 3:** Correct-response rates for the 17 knowledge items, all participants (n = 359).

Item	Knowledge Item	Correct, n (%)	Unanswered, n
K1	Distinction between extravasation and infiltration	313 (87.2)	10
K2	Definition of a vesicant agent	302 (84.1)	15
K3	Features of a high-risk extravasation agent *	86 (24.0)	18
K4	First step when extravasation is suspected	242 (67.4)	10
K5	Clinical features of INS Grade 4 *	50 (13.9)	39
K6	Finding not among the “5 P” of compartment syndrome	324 (90.3)	13
K7	Anatomical factor in dorsal-hand extravasation	274 (76.3)	15
K8	Mechanism of action of hyaluronidase	300 (83.6)	17
K9	Severity grade from which the flush-out technique is indicated	196 (54.6)	39
K10	Antidote for vasopressor extravasation	203 (56.5)	41
K11	Antidote for anthracycline extravasation	184 (51.3)	48
K12	Decision on warm versus cold application	211 (58.8)	35
K13	Maximum time window for the flush-out technique	185 (51.5)	37
K14	Indications for urgent surgical consultation *	97 (27.0)	23
K15	Surgeon’s role in perioperative contrast extravasation	301 (83.8)	27
K16	Risk factors at the peripheral IV site *	64 (17.8)	22
K17	Information that should not be documented	257 (71.6)	19

* Multiple-answer item, scored all-or-nothing (all correct options selected and no distractor selected). Unanswered items were scored as incorrect; percentages are therefore calculated from the full analytic sample (n = 359) for every item. Item K5 contained four correct options and no distractor.

**Table 4:** Total knowledge score and proportion with a high knowledge level (≥12/17) by subgroup.

Subgroup	n	Knowledge Score, Mean ± SD	*p* *	High Knowledge, n (%)	*p* ^†^
Specialty					
Orthopaedics and Traumatology	211	9.2 ± 3.5	<0.001	54 (25.6)	<0.001
Plastic, Reconstructive and Aesthetic Surgery	148	11.1 ± 3.3		73 (49.3)	
Professional seniority					
0–2 years	103	10.1 ± 3.3	0.175	36 (35.0)	0.122
3–5 years	115	10.2 ± 3.9		48 (41.7)	
6–10 years	52	10.4 ± 3.3		20 (38.5)	
≥10 years	89	9.4 ± 3.3		23 (25.8)	
Formal training					
Not received	252	9.9 ± 3.5	0.311	85 (33.7)	0.317
Received	107	10.3 ± 3.6		42 (39.3)	
Type of institution					
Other	69	9.4 ± 3.6	0.075	18 (26.1)	0.073
University/training and research hospital	290	10.1 ± 3.5		109 (37.6)	
Frequency of case exposure					
Infrequent (<a few times/month)	187	9.6 ± 3.4	0.017	58 (31.0)	0.072
Frequent (≥a few times/month)	172	10.4 ± 3.6		69 (40.1)	
Experience of surgical intervention					
Never required	86	9.6 ± 3.3	0.119	24 (27.9)	0.097
Required in at least some cases	273	10.1 ± 3.6		103 (37.7)	

* Mann–Whitney *U* test for two-group comparisons and Kruskal–Wallis test for professional seniority. ^†^ Pearson chi-square test. The university/training and research hospital category includes two participants working in city hospitals; the “Other” category includes the four participants who did not state their institution.

**Table 5:** Univariable and multivariable logistic regression for a high knowledge level (≥12/17).

Variable	Univariable OR (95% CI)	*p*	Multivariable Adjusted OR (95% CI)	*p*
Plastic surgery (ref.: Orthopaedics and Traumatology)	2.77 (1.76–4.35)	<0.001	3.00 (1.74–5.18)	<0.001
Seniority of 3–5 years (ref.: 0–2 years)	1.33 (0.76–2.30)	0.318	1.34 (0.75–2.38)	0.322
Seniority of 6–10 years (ref.: 0–2 years)	1.15 (0.57–2.29)	0.699	1.87 (0.84–4.17)	0.128
Seniority ≥10 years (ref.: 0–2 years)	0.60 (0.32–1.14)	0.119	0.89 (0.41–1.94)	0.764
Formal training received (ref.: not received)	1.30 (0.81–2.09)	0.269	0.95 (0.56–1.61)	0.851
University/training and research hospital (ref.: other)	1.90 (1.01–3.56)	0.046	1.20 (0.55–2.62)	0.645
Frequent case exposure, ≥a few times/month (ref.: infrequent)	1.54 (0.99–2.39)	0.056	0.85 (0.48–1.51)	0.579
Experience of surgical intervention (ref.: never)	1.53 (0.90–2.61)	0.118	1.29 (0.71–2.33)	0.398

OR, odds ratio; CI, confidence interval. Both the univariable and the multivariable models were fitted on the same complete-case set (n = 351; 124 events; 8 participants excluded for missing data on institution, frequency of case exposure or experience of surgical intervention), giving 15.5 events per estimated parameter. Nagelkerke R^2^ = 0.100; area under the ROC curve = 0.662; Hosmer–Lemeshow *p* = 0.616. Variance inflation factors ranged from 1.15 to 2.07, indicating no relevant collinearity. No observation was influential: the maximum Cook’s distance was 0.016, the maximum leverage was 0.067 (below the 3k/n cut-off of 0.077) and the largest absolute standardised Pearson residual was 2.29; excluding the 12 observations exceeding the liberal 4/n cut-off strengthened rather than attenuated the specialty association (adjusted OR, 4.58; 95% CI, 2.50–8.37).

## Data Availability

The datasets generated and analysed during the current study are not publicly available because participants did not consent to public data sharing but are available from the corresponding author on reasonable request.

## Supplementary Materials

The following supporting information can be downloaded at https://www.mdpi.com/article/10.3390/healthcare14213781/s1, Table S1: Full survey instrument, answer key and scoring rules; Table S1b: Item-to-domain mapping, item type and observed correct-response rate; Table S1c: Provenance of the answer key: mapping of each knowledge item to its source; Table S2: Option-level selection frequency for the four multiple-answer knowledge items; Table S3: Sensitivity analysis: multivariable model using the median score (≥10/17) as the threshold; Table S4: Correct-response rates for each knowledge item by specialty; Table S5: Correct-response rates for each knowledge item by professional seniority; Table S6: Correct-response rates for each knowledge item by prior training status; Table S7: Stability of the multivariable model across alternative thresholds for a high knowledge level. The supplementary materials additionally cite references [28,29] in Table S1c and the CROSS [30] and STROBE [31] reporting checklists.

## Abbreviations

The following abbreviations are used in this manuscript:

AUC	Area under the receiver operating characteristic curve
CI	Confidence interval
INS	Infusion Nurses Society
IQR	Interquartile range
KR-20	Kuder–Richardson Formula 20
OR	Odds ratio
SD	Standard deviation
