# Supplementary material for: Knowledge Gaps in Evidence-Based Extravasation Management: A Cross-Sectional Survey of Surgical Specialists in Türkiye
Source: Healthcare (Basel). 2026 Nov 5;14(21):3781. doi: 10.3390/healthcare14213781 (PMC13762762; doi:10.3390/healthcare14213781)
Supplement: Supplementary file 1 [file healthcare-14-03781-s001.zip › healthcare-4627653-supplementary.pdf]

Supplementary Materials

Knowledge Gaps in Evidence-Based Extravasation Management: A Cross-Sectional Survey of Surgical Specialists in Türkiye

Contents

Table S1. Full survey instrument, answer key and scoring rules
Table S1b. Item-to-domain mapping, item type and observed correct-response rate
Table S1c. Provenance of the answer key: mapping of each knowledge item to its source
Table S2. Option-level selection frequency for the four multiple-answer knowledge items
Table S3. Sensitivity analysis using the median score (≥10/17) as the threshold
Table S4. Correct-response rates for each knowledge item by specialty
Table S5. Correct-response rates for each knowledge item by professional seniority
Table S6. Correct-response rates for each knowledge item by prior training status
Table S7. Stability of the multivariable model across alternative thresholds
CROSS reporting checklist for survey studies — completed
STROBE reporting checklist for cross-sectional studies — completed

Supplementary Material

Table S1. Full survey instrument, answer key and scoring rules

Knowledge Gaps in Evidence-Based Extravasation Management: A Cross-Sectional Survey of Surgical Specialists in Türkiye

Instrument structure

The survey was administered in Turkish via an online form between 1 and 18 June 2026. It comprised 29 items in three sections: 5 items on participant characteristics (Section 1), 7 items on clinical experience, institutional infrastructure and attitudes (Section 2), and 17 knowledge items (Section 3, items 13–29 of the administered form). Only the 17 knowledge items contributed to the knowledge score. The instrument is reproduced below in English translation; the Turkish original is available from the corresponding author on request. Correct responses are marked "✓".

Scoring rules

1. Each of the 17 knowledge items contributed a maximum of 1 point, giving a total score range of 0–17.
2. Thirteen items were single-best-answer; 4 items were multiple-answer (marked "multiple-answer" below).
3. Multiple-answer items were scored all-or-nothing: an item was counted correct only if every correct option was selected and no distractor option was selected. No partial credit was given.
4. Item K5 (INS Grade 4 features) is a multiple-answer item in which all four options are correct; it therefore contained no distractor, and a correct response required selection of all four options.
5. Unanswered knowledge items were scored as incorrect.
6. The total score was dichotomised at ≥70% correct (≥12/17) to define "high knowledge level".

Section 1. Participant characteristics (items 1–5)

1. What is your specialty?

- ○ Orthopaedics and Traumatology
- ○ Plastic, Reconstructive and Aesthetic Surgery
- ○ Other: ____________

2. What is your professional seniority?

- ○ 0–2 years (Resident)
- ○ 3–5 years (Resident)
- ○ 6–10 years (Specialist)
- ○ ≥10 years (Senior Specialist / Consultant)

3. What type of institution do you work in?

- ○ University hospital
- ○ Training and research hospital
- ○ State hospital
- ○ Private hospital
- ○ Other: ____________

4. Have you previously received training on the management of vascular extravasation?

- ○ Yes, both theoretical and practical training
- ○ Yes, theoretical training only
- ○ No structured training; learned through clinical experience
- ○ No training at all

5. What are your sources of knowledge on this topic? (multiple responses permitted)

- ○ Medical school / residency education
- ○ Clinical experience
- ○ Academic articles and guidelines
- ○ Congress / symposium
- ○ Learning from colleagues
- ○ Institutional protocols

Section 2. Clinical experience, institutional infrastructure and attitudes (items 6–12)

6. How frequently do you encounter extravasation cases in your practice?

- ○ More than once per week
- ○ Once per week

- ○ A few times per month
- ○ A few times per year
- ○ Never encountered

7. In which scenario are you most frequently consulted for an extravasation case?

- ○ Peripheral intravenous chemotherapy extravasation (vesicant agent)
- ○ Non-cytotoxic agent extravasation (contrast, vasopressor, hyperosmolar solution, etc.)
- ○ Late presentation (established necrosis / eschar)
- ○ Suspected compartment syndrome
- ○ Other: ____________

8. In your typical practice, how often do extravasation cases require surgical intervention (debridement, flap, graft, etc.)?

- ○ In the majority of cases (> 50%)
- ○ In approximately half of cases (25–50%)
- ○ In a minority of cases (< 25%)
- ○ Never required

9. Where do you experience the greatest difficulty in extravasation management? (multiple responses permitted)

- ○ Late presentation due to delayed recognition of the injury
- ○ Delayed first-line intervention (e.g. flush technique)
- ○ Difficulty determining treatment strategy according to agent type
- ○ Lack of institutional protocol / guideline
- ○ Inadequate interdisciplinary coordination
- ○ Inadequate planning of flap / reconstruction options

10. Is there a written protocol / guideline for extravasation management at your institution?

- ○ Yes, exists and is actively implemented
- ○ Yes, exists but is underutilised
- ○ No institutional protocol
- ○ Unaware of whether one exists

11. Is an incident report completed after an extravasation case at your institution?

- ○ Completed for every case
- ○ Completed for serious / surgical cases
- ○ Rarely completed
- ○ No reporting system, or not used

12. Would you like to receive further training on this topic?

- ○ Yes, particularly a multidisciplinary simulation / cadaver course
- ○ Yes, a theoretical update including current guidelines
- ○ No, current knowledge is sufficient

Section 3. Knowledge items (K1–K17; items 13–29 of the administered form)

Correct responses are marked "✓". Domain headings follow the structure of the administered instrument.

A. Basic definition and classification

K1. What is the fundamental difference between extravasation and infiltration?

- ○ The two are identical concepts and may be used interchangeably
- ✓ Extravasation is leakage into surrounding tissue of an agent with tissue-damaging potential; infiltration involves an agent without such potential
- ○ Extravasation occurs only with chemotherapeutic drugs
- ○ Infiltration is arterial in origin and extravasation venous

K2. Which of the following best describes the definition of a vesicant agent?

- ○ An agent that causes only pain and oedema
- ✓ An agent capable of causing blistering, ulceration and necrosis on tissue leakage
- ○ Drugs belonging exclusively to the chemotherapy class
- ○ Any agent with a pH below 7

K3. Which of the following properties make a drug a high-risk extravasation agent?

(multiple-answer item)

- ✓ Osmolality > 600 mOsm/kg
- ✓ pH < 5 or pH > 9
- ✓ Vasoconstrictive effect (norepinephrine, dopamine, etc.)
- ○ Isotonic, neutral-pH solution

B. Clinical diagnosis and grading

K4. What is the first step to be taken when extravasation is suspected?

- ○ Immediate removal of the cannula
- ✓ Stopping the infusion and attempting aspiration while leaving the cannula in place
- ○ Initiating cold application
- ○ Direct referral to plastic surgery

K5. Which of the following are among the clinical features of Grade 4 on the INS (Infusion Nurses Society) infiltration/extravasation scale?

(multiple-answer item)

- ✓ Pale, translucent skin
- ✓ Oedema extending more than 6 inches (≈ 15 cm)
- ✓ Marked tissue oedema (deep pitting)
- ✓ Circulatory compromise and moderate-to-severe pain

K6. Which of the following is NOT among the "5 P" findings assessed when compartment syndrome is suspected at an extravasation site?

- ○ Pain
- ○ Pallor
- ○ Paresthesia
- ✓ Pruritus

K7. What is the most important anatomical factor worsening the prognosis of extravasation on the dorsum of the hand?

- ✓ Thin skin and scanty subcutaneous fat
- ○ Position of the extremity
- ○ Age of the patient
- ○ Diameter of the catheter used

C. Treatment decisions

K8. What is the mechanism of action of hyaluronidase used in the flush-out technique?

- ○ It neutralises the toxic substance
- ✓ It hydrolyses hyaluronic acid, allowing the extravasated fluid to be dispersed and resorbed more readily
- ○ It is a potent vasoconstrictor
- ○ It anaesthetises the surrounding tissue locally

K9. From which severity grade onwards is the flush-out technique considered indicated?

- ○ INS Grade 1
- ○ INS Grade 2
- ✓ INS Grade 3 and above
- ○ Grade 4 only

K10. What is the preferred pharmacological antidote in vasopressor (norepinephrine, dopamine, etc.) extravasation?

- ○ Hyaluronidase
- ○ Dexrazoxane
- ✓ Phentolamine (subcutaneous), or alternatively terbutaline
- ○ Topical DMSO

K11. Which agent is recommended as the specific antidote in extravasation of anthracyclines (epirubicin, doxorubicin)?

- ○ Hyaluronidase
- ✓ Dexrazoxane (IV)
- ○ Phentolamine
- ○ Methylprednisolone

K12. How should the decision between warm and cold application be made in vesicant agent extravasation?

○ Cold application is always recommended
○ Warm application is always recommended
✓ Cold for DNA-binding vesicants (anthracyclines); warm for vinca alkaloids and vasopressors
○ Warm/cold application has no proven effect and should not be used

D. Surgical timing and referral

K13. What is the recommended maximum time window for the flush-out technique to be effective in vesicant agent extravasation?

○ Within the first 1 hour
✓ Within the first 6 hours
○ Within the first 12 hours
○ Within the first 24 hours

K14. Which of the following clinical findings constitute an absolute indication for urgent consultation with plastic / orthopaedic surgery?

(multiple-answer item)
✓ Blistering and skin necrosis
✓ Findings of compartment syndrome (5 P)
✓ Suspected extensor tendon injury
○ Minimal erythema and oedema under 1 cm

K15. What is the role of the orthopaedic / plastic surgeon when perioperative contrast medium extravasation occurs?

○ No intervention is required because contrast medium has a neutral pH
✓ Conservative treatment (elevation, warm/cold application) is initiated; urgent fasciotomy is performed if there are findings of compartment syndrome
○ Urgent debridement is performed in every case
○ It is solely the responsibility of the radiologist and surgical disciplines are not involved

E. Risk factors, prevention and reporting

K16. Which of the following, relating to the peripheral IV injection site, most increase the risk of extravasation?

(multiple-answer item)
✓ A site close to a joint (antecubital fossa, wrist)
✓ Leaving the catheter in place for a prolonged period (> 24 h)
✓ Small-calibre veins and damaged vascular structures
○ Placement in the mid upper arm or mid forearm

K17. Which information should NOT be included in the documentation of an extravasation case?

○ Name and concentration of the drug and estimated extravasated volume
○ Time and location of the injury
○ Initial interventions performed
✓ The patient’s financial situation and insurance details

Table S1b. Item-to-domain mapping, item type and observed correct-response rate (n = 359)

Domains: A, basic definition and classification; B, clinical diagnosis and grading; C, treatment decisions; D, surgical timing and referral; E, risk factors, prevention and reporting. Multiple-answer items were scored all-or-nothing (all correct options selected and no distractor selected). Item K5 contained four correct options and no distractor.

Score distribution (n = 359)

Mean ± SD 10.00 ± 3.50; median 10 (IQR 8–13); range 0–17. Kuder–Richardson 20 = 0.795. Using the pre-specified threshold of ≥70% correct (≥12/17), 127 participants (35.4%) were classified as having a high knowledge level.

Item	Domain	Type	Content	Correct, n (%)
K1	A	Single	Distinction between extravasation and infiltration	313 (87.2)
K2	A	Single	Definition of a vesicant agent	302 (84.1)
K3	A	Multiple	Features of a high-risk extravasation agent	86 (24.0)
K4	B	Single	First step when extravasation is suspected	242 (67.4)
K5	B	Multiple	Clinical features of INS Grade 4	50 (13.9)
K6	B	Single	Finding not among the "5 P" of compartment syndrome	324 (90.3)
K7	B	Single	Anatomical factor in dorsal-hand extravasation	274 (76.3)
K8	C	Single	Mechanism of action of hyaluronidase	300 (83.6)
K9	C	Single	Grade from which flush-out is indicated	196 (54.6)
K10	C	Single	Antidote for vasopressor extravasation	203 (56.5)
K11	C	Single	Antidote for anthracycline extravasation	184 (51.3)
K12	C	Single	Warm versus cold application	211 (58.8)
K13	D	Single	Maximum time window for flush-out technique	185 (51.5)
K14	D	Multiple	Indications for urgent surgical consultation	97 (27.0)
K15	D	Single	Surgeon’s role in perioperative contrast extravasation	301 (83.8)
K16	E	Multiple	Risk factors at the peripheral IV site	64 (17.8)
K17	E	Single	Information not to be documented	257 (71.6)

Supplementary Material

Table S1c. Provenance of the answer key: mapping of each knowledge item to its source

Knowledge Gaps in Evidence-Based Extravasation Management: A Cross-Sectional Survey of Surgical Specialists in Türkiye

Each of the 17 knowledge items (K1–K17) was keyed against published guidelines and peer-reviewed literature. The "Primary source" column gives the reference on which the keyed answer was based; the "Corroborating source" column gives an additional independent source that states the same content. Verbatim supporting text is reproduced in the final column. Reference numbers refer to the reference list of the main manuscript.

Item	Keyed correct answer (abridged)	Primary source	Corroborating source	Verbatim supporting text
**K1**	Extravasation = leakage of an agent with tissue-damaging potential; infiltration = leakage of a non-damaging agent	[1, 2]	[S1] Chang & Murray 2020	"Infiltration from irritant medications causes inflammation and pain, while extravasation from vesicant medications also damages soft tissue, leading to blistering and tissue necrosis."
**K2**	A vesicant is an agent capable of causing blistering, ulceration and necrosis on tissue leakage	[9, 10]	[S2] Yellinedi et al. 2023	"Vesicants extravasation can produce blistering and severe local tissue damage." / "cytotoxic agents are classified into three groups … vesicants, irritants, and non-tissue-damaging agents."
**K3**	High-risk features: osmolality > 600 mOsm/kg; pH < 5 or > 9; vasoconstrictive effect	[11] INS Infusion Therapy Standards of Practice	[16] Hackenberg et al. 2021	"All substances with the following properties are regarded as potentially vesicant: pH <5, pH >9, >600 mOsmol/kg, vasopressors."
**K4**	Stop the infusion and attempt aspiration, leaving the cannula in place	[8, 11]	[14] Stefanos et al. 2023	"Stop the intravenous push or infusion immediately … The catheter or needle should be left in place to attempt aspiration of fluid from the extravasated area." / "Stop the infusion! … Do NOT remove cannula … Aspirate any residual drug from cannula."
**K5**	INS Grade 4: pale translucent skin; oedema > 6 inches; marked (deep pitting) oedema; circulatory compromise with moderate-to-severe pain	[11] INS Infiltration/Extravasation Scale	— (no second source; the INS scale is the sole definitional authority for this item)	Keyed directly from the four descriptors of Grade 4 in the INS Infiltration/Extravasation Scale.
**K6**	Pruritus is NOT one of the "P" findings of compartment syndrome	[3, 4]	[14] Stefanos et al. 2023	"The '6 P's' of compartment syndrome include pain, pallor, paresthesias, pulselessness, pressure, and paralysis." (Pruritus appears in no published list.)
**K7**	Thin skin and scanty subcutaneous fat over the dorsum of the hand	[3, 12]	[13] Fırat et al. 2013	"The dorsum of the hand was the most common site of the injury, causing extensor tendon exposure."
**K8**	Hyaluronidase hydrolyses hyaluronic acid, allowing extravasated fluid to disperse and be resorbed	[5, 12]	[14] Stefanos et al. 2023	"For hyperosmolar and pH-related mechanisms of injuries, hyaluronidase is most commonly used to facilitate absorption and dispersion."
**K9**	Flush-out is indicated from INS Grade 3 upwards	[11, 12, 22]	[14] Stefanos et al. 2023	"… to attempt aspiration of fluid from the extravasated area (particularly for grades 3 and 4 extravasations)."
**K10**	Phentolamine subcutaneously, or terbutaline as an alternative	[5, 20]	[14] Stefanos et al. 2023	"Historically, intradermal phentolamine has been the preferred agent for vasopressor extravasations, but frequent supply shortages have led to the emergence of terbutaline, a β2-agonist, as an acceptable alternative."
**K11**	Dexrazoxane (IV) for anthracycline extravasation	[10, 21]	[16] Hackenberg et al. 2021; [23] Mas et al. 2021	"dexrazoxane (anthracyclines)"

**K12**	Cold for DNA-binding vesicants (anthracyclines); warm for vinca alkaloids and vasopressors	[9, 21]	[13] Fırat et al. 2013; [14] Stefanos et al. 2023	"The application of a cold pack … is beneficial by decreasing the metabolism and so inactivating the destructive capacity of the extravasated agent. In contrast, cold packs are contraindicated in … vinca alkaloids … and catecholamines … so that warm packs should be applied in such agents."
**K13**	Within the first 6 hours	[12] Gault 1993	[23] Mas et al. 2021; [22] Harrold et al. 2015	"Extravasation of cytotoxic vesicant drugs is a surgical emergency (within six hours) … these incidents must be detected early (within six hours) because medical management and surgical treatment are urgent."
**K14**	Blistering/skin necrosis; findings of compartment syndrome; suspected extensor tendon injury	[3, 6]	[14] Stefanos et al. 2023; [13] Fırat et al. 2013	"If there are signs or symptoms of compartment syndrome (6 Ps), obtain a surgical consult"; "Severe injury: surgical consult for skin [necrosis]"; "treated with fasciocutaneous flaps with the tendon exposure … of the hand."
**K15**	Conservative treatment (elevation, warm/cold application); urgent fasciotomy if compartment syndrome develops	[3]; ACR Manual on Contrast Media	[15] Dien Esquivel et al. 2024	"Most patients will be managed conservatively … the most common treatment approaches include warm, cold compresses and arm elevation … patients … developing compartment syndrome requiring surgical intervention [fasciotomy]."
**K16**	Site close to a joint; catheter left in situ > 24 h; small-calibre or damaged veins	[11, 19]	[14] Stefanos et al. 2023	"The 2021 American INS Infusion Standards … recommend avoiding areas of flexion such as the antecubital fossa, wrist, ankles, or dorsum of the hand … Other infusion-specific risk factors include indwelling durations longer than 24 h." / "the infusion site close to the joint was an independent risk factor for the severity of drug extravasation."
**K17**	The patient's financial situation and insurance details should NOT be documented	[11] INS Standards — documentation requirements	—	Documentation sets specify the clinical dataset to be recorded (agent, volume, site, time, appearance, photograph, incident report); financial and insurance data form no part of it and are excluded by data-minimisation principles. This item was keyed as a face-valid distractor rather than from a single quotable sentence.

Notes

1. Items K1–K4, K6 and K8–K17 are each supported by at least one verbatim statement in a published source, reproduced above. Item K5 is keyed directly from the Grade 4 descriptors of the INS Infiltration/Extravasation Scale, which is itself the definitional authority for the item and therefore has no independent corroborating source. Item K17 was keyed as a face-valid distractor item; no published source states the exclusion explicitly.

2. For item K6 the instrument used the phrase "5 P". Published lists vary between five and six "P" findings depending on whether "pressure" (or "poikilothermia") is included. The keyed answer is unaffected, because pruritus appears in no published list; nevertheless the manuscript now refers to "the classical 'P' findings" rather than to a fixed number.

3. References 13–16 (Fırat 2013, Stefanos 2023, Dien Esquivel 2024, Hackenberg 2021) were added to the reference list of the revised manuscript in response to reviewer comments. [S1] and [S2] are cited in this table only and are listed at the end. An em dash indicates that no independent second source was identified.

References added to the main reference list in revision

Four references (nos. 13–16 of the revised reference list) were added in revision. Every other numbered source cited in Table S1c was already present in the submitted version.

13. Dien Esquivel, M.F.; Miller, E.; Bijelić, V.; Barrowman, N.; Putnins, R. CT Contrast Extravasation in Children: A Single-Center Experience and Systematic Review. Pediatr. Radiol. 2024, 54, 34–42, doi:10.1007/s00247-023-05811-6.

15. Fırat, C.; Erbatur, S.; Aytekin, A.H. Management of Extravasation Injuries: A Retrospective Study. J. Plast. Surg. Hand Surg. 2013, 47, 60–65, doi:10.3109/2000656X.2012.741065.

16. Hackenberg, R.K.; Kabir, K.; Müller, A.; Heydweiller, A.; Burger, C.; Welle, K. Extravasation Injuries of the Limbs in Neonates and Children—Development of a Treatment Algorithm. Dtsch. Arztebl. Int. 2021, 118, 547–554, doi:10.3238/arztebl.m2021.0220.

14. Stefanos, S.S.; Kiser, T.H.; MacLaren, R.; Mueller, S.W.; Reynolds, P.M. Management of Noncytotoxic Extravasation Injuries: A Focused Update on Medications, Treatment Strategies, and Peripheral Administration of Vasopressors and Hypertonic Saline. Pharmacotherapy 2023, 43, 321–337, doi:10.1002/phar.2794.

Sources cited in Table S1c only (not added to the main reference list)

S1. Chang, R.; Murray, N. A Case of Mitoxantrone Extravasation. J. Oncol. Pharm. Pract. 2020, 26, 1518–1521, doi:10.1177/1078155219893736.

S2. Yellinedi, R.; Damalachervu, M.R.; Thammineedi, S.R. Management of Epirubicin Extravasation Injuries of the Hand with Debridement and Flap Cover—A Case Series. Indian J. Plast. Surg. 2023, 56, 439–443, doi:10.1055/s-0043-1774787.

Table S2. Option-level selection frequency for the four multiple-answer knowledge items (n = 359).

Option	Status	n	%
**K3. Features of a high-risk extravasation agent**			
Osmolality > 600 mOsm/kg	Correct	193	53.8
pH < 5 or pH > 9	Correct	227	63.2
Vasoconstrictive effect (norepinephrine, dopamine, etc.)	Correct	150	41.8
Isotonic, neutral-pH solution	Distractor	18	5.0
All correct options selected and no distractor	—	86	24.0
**K5. Clinical features of INS Grade 4**			
Pale, translucent skin	Correct	121	33.7
Oedema extending more than 6 inches (≈ 15 cm)	Correct	109	30.4
Marked tissue oedema (deep pitting)	Correct	117	32.6
Circulatory compromise and moderate-to-severe pain	Correct	213	59.3
All correct options selected and no distractor	—	50	13.9
**K14. Indications for urgent surgical consultation**			
Blistering and skin necrosis	Correct	176	49.0
Findings of compartment syndrome (5 P)	Correct	312	86.9
Suspected extensor tendon injury	Correct	120	33.4
Minimal erythema and oedema under 1 cm	Distractor	14	3.9
All correct options selected and no distractor	—	97	27.0
**K16. Risk factors at the peripheral IV site**			
Site close to a joint (antecubital fossa, wrist)	Correct	178	49.6
Catheter left in place > 24 h	Correct	190	52.9
Small-calibre veins and damaged vasculature	Correct	210	58.5
Placement in the mid upper arm or mid forearm	Distractor	76	21.2
All correct options selected and no distractor	—	64	17.8

Percentages are calculated from the full analytic sample (n = 359); participants who did not answer the item are included in the denominator. Item K5 contained four correct options and no distractor.

Table S3. Sensitivity analysis: multivariable model using the median score (≥10/17) as the threshold.

Variable	Adjusted OR (95% CI)	p
Plastic surgery (ref.: Orthopaedics and Traumatology)	2.65 (1.55–4.55)	<0.001
Seniority 3–5 years (ref.: 0–2 years)	1.07 (0.60–1.90)	0.824
Seniority 6–10 years (ref.: 0–2 years)	2.00 (0.90–4.42)	0.088
Seniority ≥10 years (ref.: 0–2 years)	1.17 (0.56–2.44)	0.681
Formal training received (ref.: not received)	0.69 (0.41–1.16)	0.164
University / training and research hospital (ref.: other)	1.46 (0.72–2.95)	0.295
Frequent case exposure (ref.: infrequent)	1.19 (0.69–2.05)	0.541
Experience of surgical intervention (ref.: never)	1.29 (0.74–2.23)	0.364

n = 351; 209 events. Nagelkerke R^2 = 0.091; area under the ROC curve = 0.657. The association between plastic surgery specialty and a high knowledge level was unchanged when the median score was used instead of the pre-specified ≥70% threshold.

Table S4. Correct-response rates for each knowledge item by specialty (n = 359).

Item	Knowledge item	Orthopaedics n = 211	Plastic surgery n = 148	p	p (Holm)
K1	Distinction between extravasation and infiltration	175 (82.9)	138 (93.2)	0.004	0.052
K2	Definition of a vesicant agent	173 (82.0)	129 (87.2)	0.187	0.748
K3	Features of a high-risk extravasation agent *	39 (18.5)	47 (31.8)	0.004	0.052
K4	First step when extravasation is suspected	135 (64.0)	107 (72.3)	0.098	0.510
K5	Clinical features of INS Grade 4 *	22 (10.4)	28 (18.9)	0.022	0.244
K6	Finding not among the "5 P" of compartment syndrome	185 (87.7)	139 (93.9)	0.050	0.381
K7	Anatomical factor in dorsal-hand extravasation	160 (75.8)	114 (77.0)	0.793	0.998
K8	Mechanism of action of hyaluronidase	169 (80.1)	131 (88.5)	0.034	0.314
K9	Severity grade from which the flush-out technique is indicated	106 (50.2)	90 (60.8)	0.048	0.381
K10	Antidote for vasopressor extravasation	102 (48.3)	101 (68.2)	<0.001	0.003
K11	Antidote for anthracycline extravasation	77 (36.5)	107 (72.3)	<0.001	<0.001
K12	Decision on warm versus cold application	107 (50.7)	104 (70.3)	<0.001	0.003
K13	Maximum time window for the flush-out technique	105 (49.8)	80 (54.1)	0.423	0.998
K14	Indications for urgent surgical consultation *	53 (25.1)	44 (29.7)	0.333	0.998
K15	Surgeon’s role in perioperative contrast extravasation	171 (81.0)	130 (87.8)	0.085	0.510
K16	Risk factors at the peripheral IV site *	28 (13.3)	36 (24.3)	0.007	0.085
K17	Information that should not be documented	142 (67.3)	115 (77.7)	0.031	0.314

*Values are n (%) of all participants in the group; unanswered items were scored as incorrect. p values are from Pearson chi-square tests (Fisher exact test where expected cell counts were <5). p (Holm) denotes p values adjusted for 17 comparisons using the Holm procedure. * Multiple-answer item scored all-or-nothing.*

Table S5. Correct-response rates for each knowledge item by professional seniority (n = 359).

Item	Knowledge item	0–2 y n = 103	3–5 y n = 115	6–10 y n = 52	≥10 y n = 89	p	p (Holm)
K1	Distinction between extravasation and infiltration	92 (89.3)	101 (87.8)	42 (80.8)	78 (87.6)	0.495	1.000
K2	Definition of a vesicant agent	86 (83.5)	99 (86.1)	42 (80.8)	75 (84.3)	0.849	1.000
K3	Features of a high-risk extravasation agent *	25 (24.3)	31 (27.0)	14 (26.9)	16 (18.0)	0.463	1.000
K4	First step when extravasation is suspected	66 (64.1)	71 (61.7)	40 (76.9)	65 (73.0)	0.131	1.000
K5	Clinical features of INS Grade 4 *	16 (15.5)	19 (16.5)	6 (11.5)	9 (10.1)	0.533	1.000
K6	Finding not among the "5 P" of compartment syndrome	97 (94.2)	103 (89.6)	47 (90.4)	77 (86.5)	0.351	1.000
K7	Anatomical factor in dorsal-hand extravasation	77 (74.8)	85 (73.9)	42 (80.8)	70 (78.7)	0.718	1.000
K8	Mechanism of action of hyaluronidase	83 (80.6)	96 (83.5)	46 (88.5)	75 (84.3)	0.658	1.000
K9	Severity grade from which the flush-out technique is indicated	58 (56.3)	61 (53.0)	30 (57.7)	47 (52.8)	0.908	1.000
K10	Antidote for vasopressor extravasation	56 (54.4)	67 (58.3)	29 (55.8)	51 (57.3)	0.946	1.000
K11	Antidote for anthracycline extravasation	59 (57.3)	68 (59.1)	27 (51.9)	30 (33.7)	0.002	0.026
K12	Decision on warm versus cold application	62 (60.2)	77 (67.0)	30 (57.7)	42 (47.2)	0.042	0.668
K13	Maximum time window for the flush-out technique	49 (47.6)	69 (60.0)	29 (55.8)	38 (42.7)	0.069	0.961
K14	Indications for urgent surgical consultation *	33 (32.0)	29 (25.2)	18 (34.6)	17 (19.1)	0.119	1.000
K15	Surgeon’s role in perioperative contrast extravasation	87 (84.5)	97 (84.3)	49 (94.2)	68 (76.4)	0.050	0.745
K16	Risk factors at the peripheral IV site *	19 (18.4)	26 (22.6)	7 (13.5)	12 (13.5)	0.302	1.000
K17	Information that should not be documented	74 (71.8)	79 (68.7)	41 (78.8)	63 (70.8)	0.604	1.000

*Values are n (%) of all participants in the group; unanswered items were scored as incorrect. p values are from Pearson chi-square tests. p (Holm) denotes p values adjusted for 17 comparisons. * Multiple-answer item scored all-or-nothing.*

Table S6. Correct-response rates for each knowledge item by prior training status (n = 359).

Item	Knowledge item	Theoretical + practical, n = 70	Theoretical only, n = 37	No formal training, n = 252	p	p (Holm)
K1	Distinction between extravasation and infiltration	64 (91.4)	33 (89.2)	216 (85.7)	0.417	1.000
K2	Definition of a vesicant agent	61 (87.1)	28 (75.7)	213 (84.5)	0.289	1.000
K3	Features of a high-risk extravasation agent *	20 (28.6)	4 (10.8)	62 (24.6)	0.112	1.000
K4	First step when extravasation is suspected	51 (72.9)	21 (56.8)	170 (67.5)	0.240	1.000
K5	Clinical features of INS Grade 4 *	12 (17.1)	3 (8.1)	35 (13.9)	0.438	1.000
K6	Finding not among the "5 P" of compartment syndrome	66 (94.3)	35 (94.6)	223 (88.5)	0.226	1.000
K7	Anatomical factor in dorsal-hand extravasation	58 (82.9)	27 (73.0)	189 (75.0)	0.345	1.000
K8	Mechanism of action of hyaluronidase	57 (81.4)	28 (75.7)	215 (85.3)	0.290	1.000
K9	Severity grade from which the flush-out technique is indicated	44 (62.9)	20 (54.1)	132 (52.4)	0.297	1.000
K10	Antidote for vasopressor extravasation	45 (64.3)	21 (56.8)	137 (54.4)	0.334	1.000
K11	Antidote for anthracycline extravasation	45 (64.3)	19 (51.4)	120 (47.6)	0.048	0.761
K12	Decision on warm versus cold application	49 (70.0)	23 (62.2)	139 (55.2)	0.075	1.000
K13	Maximum time window for the flush-out technique	42 (60.0)	15 (40.5)	128 (50.8)	0.146	1.000
K14	Indications for urgent surgical consultation *	17 (24.3)	7 (18.9)	73 (29.0)	0.371	1.000
K15	Surgeon's role in perioperative contrast extravasation	62 (88.6)	30 (81.1)	209 (82.9)	0.468	1.000
K16	Risk factors at the peripheral IV site *	14 (20.0)	4 (10.8)	46 (18.3)	0.472	1.000
K17	Information that should not be documented	54 (77.1)	20 (54.1)	183 (72.6)	0.034	0.571

*Values are n (%) of all participants in the group; unanswered items were scored as incorrect. The three categories are nested within the binary "formal training" variable used in Table 4 and in the regression model: "formal training received" (n = 107) comprises the first two categories. p (Holm) denotes p values adjusted for 17 comparisons. * Multiple-answer item scored all-or-nothing.*

Supplementary Material

Table S7. Stability of the multivariable model across alternative thresholds for a high knowledge level

Knowledge Gaps in Evidence-Based Extravasation Management: A Cross-Sectional Survey of Surgical Specialists in Türkiye

Binary logistic regression (Enter method) was repeated with the high-knowledge outcome defined at four alternative thresholds spanning the plausible range around the median (10/17) and the threshold used in the main analysis (12/17). All models were fitted on the same complete-case set (n = 351) with identical covariates and reference categories. Adjusted odds ratios with 95% confidence intervals are shown.

**Covariate (reference category)**	**≥10/17 (59%)**	**≥11/17 (65%)**	**≥12/17 (71%)**	**≥13/17 (76%)**
**Participants with high knowledge, n**	**209**	**165**	**124**	**89**
Plastic surgery (ref. orthopaedics)	2.65 (1.55–4.55)<br>p = <0.001	2.35 (1.40–3.94)<br>p = 0.001	3.00 (1.74–5.18)<br>p = <0.001	3.33 (1.82–6.11)<br>p = <0.001
Seniority 3–5 years (ref. 0–2)	1.07 (0.60–1.90)<br>p = 0.824	1.25 (0.71–2.19)<br>p = 0.435	1.34 (0.75–2.38)<br>p = 0.322	1.01 (0.54–1.89)<br>p = 0.969
Seniority 6–10 years (ref. 0–2)	2.00 (0.90–4.42)<br>p = 0.088	2.17 (1.00–4.71)<br>p = 0.051	1.87 (0.84–4.17)<br>p = 0.128	1.67 (0.70–4.00)<br>p = 0.247
Seniority ≥10 years (ref. 0–2)	1.17 (0.56–2.44)<br>p = 0.681	1.14 (0.55–2.37)<br>p = 0.731	0.89 (0.41–1.94)<br>p = 0.764	0.71 (0.30–1.69)<br>p = 0.440
Formal training received (ref. none)	0.69 (0.41–1.16)<br>p = 0.164	0.84 (0.50–1.39)<br>p = 0.491	0.95 (0.56–1.61)<br>p = 0.851	1.16 (0.66–2.03)<br>p = 0.611
University / training and research hospital (ref. other)	1.46 (0.72–2.95)<br>p = 0.295	1.01 (0.50–2.05)<br>p = 0.981	1.20 (0.55–2.62)<br>p = 0.645	1.25 (0.52–3.04)<br>p = 0.620
Case exposure ≥ a few times per month (ref. less often)	1.19 (0.69–2.05)<br>p = 0.541	1.49 (0.87–2.56)<br>p = 0.144	0.85 (0.48–1.51)<br>p = 0.579	0.68 (0.36–1.29)<br>p = 0.238
Any experience of surgical intervention (ref. none)	1.29 (0.74–2.23)<br>p = 0.364	1.22 (0.70–2.12)<br>p = 0.476	1.29 (0.71–2.33)<br>p = 0.398	1.40 (0.71–2.74)<br>p = 0.333
**Nagelkerke R^2**	**0.091**	**0.089**	**0.100**	**0.102**
**Area under the ROC curve**	**0.657**	**0.651**	**0.662**	**0.669**

Specialty was the only covariate significantly associated with a high knowledge level, and it remained so at every threshold examined (adjusted OR 2.35–3.33; p ≤ 0.001 throughout). No other covariate reached statistical significance at any threshold. Model discrimination and explanatory power were modest and essentially unchanged across thresholds (Nagelkerke R^2 0.089–0.102; AUC 0.651–0.669). The conclusions of the multivariable analysis are therefore not dependent on the particular cut-off used to dichotomise the knowledge score.

CROSS reporting checklist

Knowledge Gaps in Evidence-Based Extravasation Management: A Cross-Sectional Survey of Surgical Specialists in Türkiye

Completed according to Sharma A, Minh Duc NT, Luu Lam Thang T, et al. A Consensus-Based Checklist for Reporting of Survey Studies (CROSS). J Gen Intern Med 2021;36:3179–3187. Item numbering follows the published checklist; the right-hand column gives the location in the revised manuscript.

Item	Topic	Reported in the revised manuscript
**Title and abstract**		
1a	Survey design identified in title/abstract	Title; Abstract, first line ("cross-sectional web-based survey")
1b	Structured abstract	Abstract (Background/Objectives, Methods, Results, Conclusions)
**Introduction**		
2	Background and rationale	Section 1, paragraphs 1–4
3	Purpose / aim	Section 1, final paragraph
**Methods — study design**		
4	Study design named	Section 2, opening paragraph; Section 2.2
**Methods — data collection**		
5a	Structure of the questionnaire	Section 2.1; Table S1
5b	Target population, validity, reliability, scoring, sources	Section 2.1 (expert review, KR-20 = 0.795); Section 2.5 (scoring); Tables S1, S1b, S1c
5c	Pretesting / pilot testing	Section 2.1 — explicitly reported as not performed; repeated in Discussion (Limitations)
5d	Full questionnaire provided	Table S1 (full bilingual instrument with answer key)
**Methods — sample characteristics**		
6a	Study population, setting, eligibility criteria	Section 2.2
6b	Sampling technique	Section 2.2 (convenience and snowball sampling); Discussion (Limitations)
6c	Sample size and how determined	Section 2.3 — no a priori calculation; approximate detectable-effect analysis reported instead
6d	Representativeness of the sample	Section 2.2; Discussion (Limitations and generalisability)
**Methods — survey administration**		
7a	Mode of administration and contact	Section 2.2 (Google Forms; association e-mail lists and instant-messaging groups)
7b	Recruitment timeframe	Section 2.2 (1–18 June 2026); Figure 1
7c	Safeguards against duplicate participation	Section 2.2 (single-response restriction); Discussion (Limitations — residual risk stated)
**Methods — study preparation**		
8	Pre-survey preparation	Section 2.1 (literature review, guideline-based answer key, expert review); Table S1c
**Methods — ethical considerations**		
9a	Ethical approval and informed consent	Institutional Review Board Statement; Informed Consent Statement
9b	Anonymity, confidentiality and data protection	Informed Consent Statement; Data Availability Statement
**Methods — statistical analysis**		
10a	Statistical methods and software	Section 2.4 (IBM SPSS Statistics v26.0)
10b	Modification of variables	Section 2.4 (dichotomisation of training, institution, case exposure); Section 2.5; Table 5
10c	Missing data — rate, mechanism and handling	Section 2.5 (428 blank responses; 78/359 participants, 21.7%); Table 3 ("Unanswered, n"); Figure 1; Table 5 footnote (complete-case, n = 351)
10d	Non-response error	Section 2.2 (denominator unknown, response rate not calculable); Discussion (Limitations)
10e	Loss to follow-up	Not applicable — single-contact cross-sectional survey
10f	Weighting / propensity adjustment	Not applicable — no weighting was applied; stated in Discussion (Limitations)
10g	Sensitivity analyses	Section 2.5; Section 3.7; Table S3; Table S7 (four thresholds)
**Results — respondent characteristics**		
11a	Numbers at each stage, flow diagram	Figure 1; Section 2.2
11b	Reasons for non-participation / exclusion	Figure 1 (1 outside target specialties; 3 specialty not stated)
11c	Response rate and its calculation	Section 2.2 and Figure 1 — explicitly reported as not calculable (denominator unknown)
11d	Unique visitors, view / participation / completion rates	Not available — the survey platform did not record views or partial entries; stated in Section 2.2

**Results — descriptive and main findings**		
12	Participant characteristics and outcomes	Section 3.1–3.3; Tables 1 and 2
13a	Unadjusted and adjusted estimates with 95% CIs and p-values	Table 4; Table 5; Tables S4–S6 (with Holm-adjusted p values)
13b	Model building, fit and assumptions	Section 2.5; Table 5 footnote (events per parameter, VIF, Cook's distance, leverage, residuals, Hosmer–Lemeshow, Nagelkerke R^2, AUC)
13c	Sensitivity analyses reported	Section 3.7; Table S3; Table S7
**Discussion**		
14	Limitations	Discussion, final paragraph
15	Interpretation	Discussion, paragraphs 1–7; Section 5
16	Generalisability	Section 2.2; Discussion (Limitations); Section 5
**Other sections**		
17	Role of the funding source	Funding statement (no external funding)
18	Conflict of interest	Conflicts of Interest statement
19	Acknowledgements	Author Contributions; no separate acknowledgements

Items 10e, 10f and 11d are reported as not applicable or not available, with the reason given in the manuscript rather than left blank.

STROBE reporting checklist — cross-sectional studies

Knowledge Gaps in Evidence-Based Extravasation Management: A Cross-Sectional Survey of Surgical Specialists in Türkiye

Completed according to the STROBE Statement checklist for cross-sectional studies (von Elm E, Altman DG, Egger M, Pocock SJ, Gøtzsche PC, Vandenbroucke JP; STROBE Initiative). The right-hand column gives the location of each item in the revised manuscript.

Item	Recommendation	Reported in the revised manuscript
**Title and abstract**		
1a	Study design named in title or abstract	Title; Abstract, first sentence of Methods ("cross-sectional web-based survey")
1b	Balanced, informative abstract	Abstract (Background/Objectives, Methods, Results, Conclusions)
**Introduction**		
2	Background / rationale	Section 1, paragraphs 1–4
3	Objectives and prespecified hypotheses	Section 1, final paragraph (primary aim; specialty comparison stated as a secondary objective)
**Methods**		
4	Key design elements presented early	Section 2, opening paragraph
5	Setting, locations and relevant dates	Section 2.2 — recruitment through the e-mail lists and instant-messaging groups of two national specialty associations in Türkiye, 1–18 June 2026
6a	Eligibility criteria and selection of participants	Section 2.2; Figure 1
7	Variables: outcomes, predictors, confounders	Section 2.4 and Section 2.5 (knowledge score 0–17; high knowledge level ≥12/17; covariates specialty, seniority, training, institution, case exposure, surgical experience)
8	Data sources and measurement	Section 2.1 (instrument development and expert review); Section 2.2 (Google Forms administration); Tables S1, S1b and S1c (instrument, mapping and answer-key provenance)
9	Efforts to address sources of bias	Section 2.2 (single-response restriction); Section 2.6 (self-reported data); Discussion, Limitations (selection, recall and social-desirability bias; unverifiable credentials; possible use of external resources)
10	How the study size was determined	Section 2.3 — no a priori sample-size calculation; recruitment open to all eligible clinicians during the study period; an approximate detectable-effect analysis is reported instead (smallest detectable absolute between-specialty difference ≈ 14.8 percentage points at $n_1 = 211$ and $n_2 = 148$, two-sided $\alpha = 0.05$, 80% power, assumed baseline 50%)
11	Handling of quantitative variables and groupings	Section 2.4 (dichotomisation of training, institution and case exposure, with reasons); Section 2.5 (dichotomisation of the knowledge score at ≥12/17 and rationale); Table 5 (reference categories)
12a	Statistical methods, including control of confounding	Section 2.4; Section 2.5 (multivariable binary logistic regression, Enter method)
12b	Methods for subgroups and interactions	Section 2.4 — item-level comparisons across specialty, seniority and training status pre-specified as exploratory and Holm-adjusted; no interaction terms were fitted
12c	Handling of missing data	Section 2.5 — unanswered knowledge items scored as incorrect (428 blank responses; 78/359 participants, 21.7%); complete-case analysis for the multivariable model (n = 351); Table 3 "Unanswered, n" column; Figure 1
12d	Methods accounting for the sampling strategy	Not applicable — convenience and snowball sampling without weighting; stated in Section 2.2 and the Limitations
12e	Sensitivity analyses	Section 2.5; Section 3.7; Table S3 (median threshold); Table S7 (four thresholds)
**Results**		
13a	Numbers at each stage	Figure 1 — 363 recorded, 4 excluded, 359 analysed, 351 complete cases
13b	Reasons for non-participation / exclusion	Figure 1 (1 outside target specialties; 3 specialty not stated). The number invited or reached is unknown, so non-participation before entry cannot be quantified; stated in Section 2.2
13c	Flow diagram	Figure 1
14a	Participant characteristics and potential confounders	Sections 3.1–3.3; Tables 1 and 2
14b	Number of participants with missing data per variable	Section 2.5; Table 3 ("Unanswered, n" per item); Table 5 footnote (institution n = 4, case exposure n = 1, surgical experience n = 4)
15	Outcome data	Section 3.4 (item-level correct-response rates, Table 3); Section 3.5 (total score; 127/359, 35.4%, with high knowledge)
16a	Unadjusted and adjusted estimates	Table 5 (univariable and adjusted odds ratios with 95% CIs, reference categories

	with precision	stated); Table 4; Tables S4–S6
16b	Category boundaries for categorised continuous variables	Section 2.5 and Table 4 (≥12/17 for the binary outcome; ≥10/17 median threshold in sensitivity analysis); Table S7 (≥10, ≥11, ≥12, ≥13)
16c	Translation of relative risk into absolute risk	Not applicable — no time-dependent risk estimates; absolute correct-response proportions are reported alongside odds ratios
17	Other analyses	Section 3.7 (sensitivity analyses); Sections 3.8–3.10 and Tables S4–S6 (exploratory subgroup comparisons with Holm-adjusted p values); Table 5 footnote (collinearity and influential-observation diagnostics)
**Discussion**		
18	Key results	Discussion, paragraph 1; Section 5
19	Limitations	Discussion, final paragraph
20	Interpretation	Discussion, paragraphs 2–7 — framed cautiously, with multiplicity and the modest explanatory power of the model acknowledged
21	Generalisability	Section 2.2; Discussion, Limitations; Section 5
**Other information**		
22	Funding	Funding statement — no external funding

Items 12d and 16c are reported as not applicable, with the reason stated rather than left blank. Item 10 is reported as a retrospective detectable-effect analysis; no a priori sample-size calculation was performed, and no such calculation is claimed.
